# The Relationship between Depression and COVID-19 Vaccine Uptake and Intention among Korean Adults: The 2021 Community Health Survey

**DOI:** 10.3390/healthcare11212809

**Published:** 2023-10-24

**Authors:** Nan Young Kim, Hae Ran Kim

**Affiliations:** 1 Department of Nursing, Graduate School, Chosun University, Gwangju 61452, Republic of Korea; ny08222@naver.com; 2 Department of Nursing, Chosun University Hospital, Gwangju 61453, Republic of Korea; 3 Department of Nursing, College of Medicine, Chosun University, Gwangju 61452, Republic of Korea

**Keywords:** COVID-19, depression, vaccine, health survey

## Abstract

Individuals with depressive symptoms are vulnerable to COVID-19 infection and mortality; therefore, their vaccination status must be investigated to reduce these rates. This study investigated the association between depressive symptoms and COVID-19 vaccine uptake and intention and identified the reasons for vaccine avoidance. Data were collected from the 2021 Korea Community Health Survey and analyzed using logistic regression. A statistically significant association was found between the presence of depressive symptoms and no COVID-19 vaccine uptake. Among individuals who reported experiencing depression, 4.6% were unvaccinated and 7.7% reported no intention to get vaccinated. Among the unvaccinated, the adjusted odds ratio (aOR) for depression was 1.53 (95% confidence interval: 1.45–1.61) compared to the vaccinated. Similarly, a significant association was observed between depressive symptoms and no intention to get vaccinated. In the group with no vaccine intention, the aOR for depression was 2.06 (95% CI: 1.86–2.27) compared to the group with vaccine intention. Furthermore, “health-related reasons” and “concerns about side effects or past experience of side effects” accounted for 89% of the reasons for vaccine avoidance among individuals with depression. Therefore, it is important to provide tailored information and develop programs to increase awareness and promote COVID-19 vaccine uptake among these individuals.

## 1. Introduction

Coronavirus Disease 2019 (COVID-19) is an acute respiratory infectious disease caused by the SARS-CoV-2 virus. The World Health Organization (WHO) declared it a “Public Health Emergency of International Concern” and declared it a pandemic in March 2020. As of November 2021, there have been over 260 million confirmed COVID-19 cases worldwide, resulting in more than 5.3 million deaths, and in South Korea, over 470,000 confirmed cases have led to more than 3000 cumulative deaths [1].

According to statistics from the Organization for Economic Cooperation and Development (OECD) for 2020, South Korea is facing a serious situation in which approximately 4 out of 10 individuals experience depression or depressive symptoms. As of 2020, the prevalence of depression in South Korea was 36.8%—the highest among OECD countries. Government measures for COVID-19 prevention, such as strict restrictions on personal gatherings, hobbies, and outdoor activities in accordance with preventive guidelines, had a negative impact on individuals’ mental well-being. This led to the emergence of the new term “Corona Blues” [2]. In fact, according to the “COVID-19 National Mental Health Survey” conducted by the Ministry of Health and Welfare in 2021, depression scores increased by more than two-fold, and the population at risk of depression was found to have increased approximately six-fold compared to 2018 [3].

In South Korea, to prevent the spread of COVID-19, measures such as mandatory mask wearing, emphasizing hand hygiene, and implementing ongoing social distancing were strengthened [4]. With the prolonged prevalence of COVID-19, vaccination emerged as the most effective means of preventing its transmission and spread [5]. As of November 2021, based on the timing of the implementation of the 2021 Community Health Survey, the domestic COVID-19 vaccination status reported a first-dose coverage of 81.7% and a full vaccination coverage of 78.1% [6].

Recent international studies have reported the need to prioritize vaccination for patients with mental health issues to reduce the spread of COVID-19 [7,8]. Previous studies have indicated that individuals with mental health problems, including depression, may face an elevated risk of COVID-19 infection-related mortality, hospitalization, and intensive care unit admission [9,10]. Target populations with mental health problems may be exposed to various factors that increase the risk of COVID-19 infection or worsen outcomes, such as difficulties in assessing health information and adhering to preventive behaviors, limited access to medical services, being homeless or living in high-risk environments, and having a high prevalence of underlying conditions related to cardiovascular diseases, cancer, and chronic obstructive pulmonary disease [11]. Despite the identification of these vulnerability factors, vaccination outcomes for individuals with mental health problems for COVID-19 prevention have not been systematically investigated [11]. Therefore, it is essential to assess vaccination rates among individuals with mental health issues residing in the community as it is necessary to reduce COVID-19 infection and mortality rates.

Recent preliminary research has reported an association between depression and COVID-19 vaccination. According to a study conducted in 2022, individuals with depression were more likely to accept misinformation regarding COVID-19 vaccines [12]. Those who accepted false health information were found to have a lower likelihood of being vaccinated and a lack of intention to be vaccinated. Another preliminary study reported that fewer than half of individuals with depression expressed hesitation toward COVID-19 vaccination, and the severity of social anxiety and depression appeared to contribute to this hesitancy [13]. In previous research on vaccine hesitancy among individuals with severe mental disorders, including depression [14], community-dwelling patients showed higher levels of hesitancy than hospitalized patients. This was attributed to limited opportunities for education on the importance and safety of vaccines and a potential lack of awareness regarding the efficacy and safety of vaccines among individuals with mental health conditions.

However, despite a consistent increase in the number of people reporting depression following COVID-19 in South Korea, research on COVID-19 vaccination and related intentions remains limited. Therefore, this study aims to investigate the relationship between depression and COVID-19 vaccination as well as vaccination intentions using raw data from the 2021 Community Health Survey, building upon the findings of previous preliminary research. Additionally, it seeks to understand the reasons for vaccine hesitancy among individuals with depression who do not intend to be vaccinated. This research is intended to provide foundational data for the development of strategies to effectively address infectious diseases among vulnerable individuals with mental health issues.

This study investigates the relationship between depression in adults, COVID-19 vaccination, and vaccination intentions. The specific objectives are as follows: to ascertain the subjects’ general characteristics, prevalence of depression, vaccination status, and vaccination intentions; to determine the association between depression status and COVID-19 vaccination and vaccination intentions among the subjects; and to identify the reasons for vaccine hesitancy among subjects with depression who lack vaccination intentions.

## 2. Methods

### 2.1. Study Design and Data Source

This secondary data analysis study aimed to investigate the relationship between depression, COVID-19 vaccination, and vaccination intentions in Korean adults using data from the 2021 Community Health Survey. This study utilized raw data from the Community Health Survey collected by the Korea Disease Control and Prevention Agency from August to October 2021. The Community Health Survey has been conducted annually since 2008 by public health centers nationwide with the aim of establishing and evaluating regional health and medical plans, standardizing survey procedures, and generating comparable regional health statistics. The sampling process for this survey involved the following steps. First, a sampling frame was created by linking the population data from the Ministry of Interior and Safety’s resident registration data and the Ministry of Land, Infrastructure, and Transport’s housing data. Second, the initial sampling was conducted in proportion to the probability of selection, considering the number of households by housing type within townships or neighborhoods/villages as the basis. Third, after identifying the number of households in the selected townships, neighborhoods, or villages as sampling points, a second-stage sample was drawn using a systematic sampling method. Trained surveyors conducted one-on-one interviews (electronic questionnaire surveys) using laptops equipped with survey software during direct visits to sampled households. Due to COVID-19, the surveyors adhered to infection control guidelines, including wearing masks, hand sanitization, temperature checks, and maintaining physical distancing when conducting household visits. Further details are available on the Community Health Survey website (http://chs.kdca.go.kr/ accessed 20 April 2023). Of the 900 people living in each of the 255 health centers, 229,242 adults participated in the 2021 KCHS.

### 2.2. Variables

All categories of research tools and analysis methods used in this study followed the guidelines provided in the Community Health Survey manual available on the survey website. The Community Health Survey uses the Patient Health Questionnaire-9 (PHQ-9). It consists of nine items that ask, “Over the past two weeks, how often have you been bothered by any of the following symptoms?” These items include “Little interest or pleasure in doing things”, “Feeling down, depressed, or hopeless”, “Trouble falling or staying asleep, or sleeping too much”, “Feeling tired or having little energy”, “Poor appetite or overeating”, “Feeling bad about yourself”, “Trouble concentrating on things, such as reading the newspaper or watching television”, “Moving or speaking so slowly that other people could have noticed”, and “Thoughts that you would be better off dead”. The respondents could choose one of four options for each item: “Not at all”, “Several days”, “More than half the days”, or “Nearly every day”. Referring to previous research, a score of 10 or more for the sum of the item scores was defined as indicative of depression [15].

COVID-19 vaccination status was determined based on the question “Have you ever received a COVID-19 vaccine?” Participants who answered “Yes” were categorized as having received the vaccination, whereas those who answered “No” were classified as not having received it.

COVID-19 vaccination intention was assessed using the item “Do you intend to receive a COVID-19 vaccine?” The participants responded by selecting one of the following options: “Strongly agree”, “Agree”, “Disagree”, or “Strongly disagree”. “Strongly agree” and “Agree” were classified as having the intention to receive the vaccine, while “Disagree” and “Strongly disagree” were classified as not having the intention to receive the vaccine.

The reasons for COVID-19 vaccination avoidance were determined using the item “If you have not received a COVID-19 vaccine, what is the primary reason for not intending to receive it?” among the participants who answered “No” to the question “Have you ever received a COVID-19 vaccine?” The item included five options, and the participants could select only one: “Because I think it is ineffective for prevention”, “Due to concerns about side effects or past experiences of side effects”, “Because I do not trust the government’s vaccination policy”, “Because I do not trust vaccine safety management by pharmaceutical companies and vaccination centers”, and “Due to health reasons”.

### 2.3. Covariate

The general characteristics considered in this study included sex, age, education level, household type, and monthly income. Sex was categorized as male and female, while age was divided into 19–29 years, 30–49 years, 50–64 years, and 65 years and older. Education level was classified as middle school graduation or below, high school graduation, or college graduation or above. Household type was categorized as living alone, living with a spouse, or other. Monthly income was classified as high, upper-middle, lower-middle, or low.

### 2.4. Statistical Data Analysis

SAS (version 9.4, SAS Institute, Cary, NC, USA) was used for data analysis in this study. Descriptive statistics were used to calculate frequencies and percentages to determine the participants’ general characteristics, depression prevalence, vaccination status, and vaccination intent. Multiple logistic regression analysis was used to examine the relationship between depression, COVID-19 vaccination status, and intent, adjusting for potential confounders. General characteristic variables were used as covariates. Descriptive statistics were also employed to calculate frequencies and percentages to determine the reasons for COVID-19 vaccination avoidance among participants with PHQ-9 scores of 10 or higher and no intent to receive the vaccine.

## 3. Results

The total number of respondents was 229,242, with 104,498 men (45.6%) and 124, 744 women (54.4%). The largest age group was 65 years or older, with 74,492 individuals (32.5%). In terms of education, the majority had completed high school, with 77,097 individuals included (33.7%). Regarding household type, 71,388 (31.2%) were living with a spouse and 38,809 (16.9%) were living alone. Monthly income showed that the high-income group was the largest, comprising 80,775 individuals (35.3%). Among the respondents, 7745 (3.4%) reported depression with a PHQ-9 score of 10 or higher, whereas 221,107 (96.6%) did not report depression, with a score below 10. In the context of COVID-19 vaccination, 188,068 individuals (82.0%) received the vaccine, while 41,469 (18.0%) did not (Table 1).

The logistic regression analysis conducted to examine the association between depression and COVID-19 vaccination revealed a statistically significant relationship. PHQ-9 scores of 10 or higher were observed in 3.1% of the vaccinated group and 4.6% of the non-vaccinated group (*p* < 0.001). In comparison to the vaccinated group, the adjusted odds ratio (aOR) for depression in the non-vaccinated group was 1.53 (95% confidence interval [CI] = 1.45–1.61; Table 2).

The association between depression and COVID-19 vaccination intention in individuals who had not yet been vaccinated against COVID-19 was statistically significant. PHQ-9 scores of 10 or higher were reported in 3.9% of the group with vaccination intent and 7.7% of the group without intent (*p* < 0.001). In comparison to the group with vaccination intent, the adjusted odds ratio (aOR) for depression in the group without intent was 2.06 (95% CI = 1.86–2.27; Table 3).

Among individuals with depression and no vaccination intent (n = 610), the reasons for avoiding COVID-19 vaccination were as follows: “Due to health reasons” for 298 individuals (48.9%), “Concerns about side effects or past experiences of adverse reactions” for 214 individuals (35.1%), “Lack of trust in the government’s vaccination policies” for 26 individuals (4.3%), “Belief that there is no preventive effect” for 18 individuals (2.9%), and ”Lack of trust in vaccine safety management by pharmaceutical companies and vaccination institutions” in 14 (2.3%) individuals (Table 4).

## 4. Discussion

This study used raw data from the 2021 Community Health Survey to analyze the relationships among depression, COVID-19 vaccination, and vaccination intent among Korean adults. Additionally, this study aimed to identify the reasons why individuals with depression avoid vaccination.

According to the research findings, among those who received the COVID-19 vaccine, 3.1% had depressive symptoms. However, among those who had not received the COVID-19 vaccine, 4.6% had depressive symptoms. This indicates that adults with depression are more likely to refrain from receiving the COVID-19 vaccine than those without depressive symptoms. Similarly, a study on the association between mental health symptoms and COVID-19 vaccination among U.S. adults reported that the proportion of individuals who received at least one dose of the vaccine was lower in adults with depression (37.7%) than in those without mental health symptoms (52.9%) [16]. Another study confirmed that individuals with chronic mental disorders had lower COVID-19 vaccination rates [17].

Individuals with mental health issues, such as depression, are more likely to be significantly affected by emotional responses to COVID-19 and have a higher likelihood of reacting sensitively to stress than those without depression; consequently, they have a known risk of worsening or relapse of pre-existing mental health conditions [18].

Examining the analysis results regarding depression and COVID-19 vaccination intention among the unvaccinated population, we found that among those with vaccination intention, the proportion of individuals exhibiting depressive symptoms was 3.9%, whereas among those without intention, the proportion of individuals exhibiting depressive symptoms was 7.7%. Furthermore, unvaccinated individuals were more likely to report depression compared to vaccinated ones. In a study investigating COVID-19 vaccination intention in patients with mental disorders in India, it was reported that only 54.5% of patients with mental disorders expressed an intent to receive the vaccine, while 37.8% declined vaccination [19]. A study on COVID-19 vaccination in Japan revealed that individuals experiencing depression and anxiety were more likely to have difficulty deciding their vaccination status [20]. This aligns with the results of our study, indicating a close relationship between depression and COVID-19 vaccination intention. Individuals experiencing depression may hesitate to get vaccinated due to a lack of energy and motivation, and those with suicidal thoughts may struggle to express minimal motivation or fail to recognize the importance of vaccination [21]. To increase the COVID-19 vaccination rate among individuals with depression, accurate information must be provided to alleviate vaccine-related anxiety and build trust. In addition, community-level vaccination promotion and related programs should be designed to provide appropriate motivation.

Approximately 90% of individuals with depression and no intention of getting vaccinated responded that they avoided vaccination due to “health reasons” and “concerns about side effects or past experiences of side effects”. According to previous studies, individuals with depressive symptoms tend to have higher levels of concern about vaccine side effects and uncertainty regarding vaccine efficacy than those without such symptoms [16]. These results suggest that individuals with depressive symptoms may experience heightened anxiety and concern about the side effects of vaccines. This can be addressed through systematic community programs. For instance, a previous study conducted in 2023 reported that providing community-level education programs encompassing COVID-19-related symptoms, transmission, prevention guidelines, vaccine efficacy, and personal health management to African American and Hispanic individuals with mental illnesses in the United States effectively increased COVID-19 vaccination rates [22]. Therefore, community nurses should develop COVID-19 vaccination-related educational programs and, with systematic support from the community, utilize various media such as the Internet and mobile phones to make them easily accessible to individuals with depressive symptoms. This aimed to enhance COVID-19 vaccination rates.

This study has several limitations. First, it was a secondary data analysis study based on raw data from the 2021 Community Health Survey, which limits the generalizability of the results. Second, the data represent the survey results from August to October 2021, reflecting a specific period rather than the overall course of the COVID-19 pandemic. Therefore, it is necessary to compare and analyze research results from various time points to complement this study. Third, depression was assessed using the PHQ-9 questionnaire, which is a self-report method different from a clinical diagnosis and may have the potential for social desirability bias, which could influence socially desirable responses. Finally, the epidemiological data used in this study pertain to the year 2021. As a result, there may be concerns regarding the current relevance of the study’s findings based on these data. It is essential for future research to take into consideration the additional confounding factors, as mentioned in recent papers, that can induce depression and anxiety beyond COVID vaccination [23].

## 5. Conclusions

This study confirmed a significant association between depression, COVID-19 vaccination, and intention to vaccinate. Therefore, it is necessary to emphasize the importance of vaccination in individuals with depression, develop specific strategies to assist in making informed vaccination decisions, and increase vaccination intentions and actual vaccination rates. Additionally, at the government and community levels, motivation programs should be developed to enhance the willingness of individuals with depression to be vaccinated, and various improvement measures should be explored. Furthermore, it can be emphasized that patients with depression visiting hospitals require professional guidance, through collaboration between healthcare professionals such as doctors and nurses as well as the local community, to emphasize the importance and safety of vaccination.

Since this study focused solely on the mental disorder of depression, future research should investigate COVID-19 vaccination in relation to various mental disorders. Subsequent studies should conduct specific inquiries and analyses regarding vaccination status, intention, and reasons for avoidance by considering each mental disorder. This will promote a deeper understanding and enable the development of more accurate and tailored policies and intervention strategies to effectively address the interaction between mental health issues and COVID-19 vaccination.

## Figures and Tables

**Table 1:** General characteristics of the participants.

Characteristics	*N*	%
Sex		
Male	104,498	45.6
Female	124,744	54.4
Age (years)		
19–29	24,695	10.8
30–49	61,269	26.7
50–64	68,786	30.0
65≤	74,492	32.5
Education level		
≤Middle school	75,492	32.9
High school	77,097	33.7
≥College	76,519	33.4
Family type		
Living with spouse	71,388	31.2
Living alone	38,809	16.9
Others	119,043	51.9
Monthly income		
High	80,775	35.3
Middle-high	43,572	19.0
Middle-low	62,411	27.2
Low	42,484	18.5
Depression (PHQ-9)		
<10	221,107	96.6
≥10	7745	3.4
COVID-19 Vaccination status		
No	41,169	18.0
Yes	188,068	82.0

PHQ = Patient Health Questionnaire-9; COVID-19 = coronavirus disease-19. Data are expressed as unweighted numbers and weighted percentages.

**Table 2:** The relationship between depression and COVID-19 vaccination.

Characteristics	COVID-19 Vaccinated Persons		COVID-19 Unvaccinated Persons		*p*
	*N*	%	*N*	%	
Depression (PHQ-9)					<0.001
No (<10)	181,945	96.6	39,157	95.3	
Yes (≥10)	5825	3.1	1920	4.6	
aOR (95% CI)	1.00		1.53 (1.45–1.61)		

aOR = adjusted odds ratio; CI = confidence interval; PHQ = Patient Health Questionnaire-9; COVID-19 = coronavirus disease-19. Data are expressed as unweighted numbers and weighted percentages.

**Table 3:** The relationship between depression and the intention to vaccinate among COVID-19 unvaccinated persons.

Characteristics	Intention to Vaccinate against COVID-19		No Intention to Vaccinate against COVID-19		*p*
	*N*	%	*N*	%	
Depression (PHQ-9)					<0.001
No (<10)	31,911	96.0	7226	92.2	
Yes (≥10)	1308	3.9	610	7.7	
aOR (95% CI)	1.00		2.06 (1.86–2.27)		

aOR = adjusted odds ratio; CI = confidence interval; PHQ = Patient Health Questionnaire-9; COVID-19 = coronavirus disease-19. Data are expressed as unweighted numbers and weighted percentages. All missing values are included.

**Table 4:** Reasons for avoiding COVID-19 vaccination among those with depression (N = 610).

Characteristics	Depression (PHQ-9)		*p*
	Yes		
	*N*	%	
What are the main reasons for the respondents indicating a lack of intention to receive COVID-19-preventive vaccinations?			<0.001
Belief that there is no preventive effect.	18	2.9	
Concerns about side effects or past experiences of adverse reactions.	214	35.1	
Lack of trust in the government’s vaccination policies.	26	4.3	
Lack of trust in vaccine safety management by pharmaceutical companies and vaccination institutions.	14	2.3	
Due to health reasons.	298	48.9	
No response	40	6.5	

PHQ = Patient Health Questionnaire-9; COVID-19 = coronavirus disease-19. Data are expressed as unweighted numbers and weighted percentages.

## Data Availability

All data are available from the corresponding author upon request.
